# NKX2-8/PTHrP Axis-Mediated Osteoclastogenesis and Bone Metastasis in Breast Cancer

**DOI:** 10.3389/fonc.2022.907000

**Published:** 2022-05-30

**Authors:** Ainiwaerjiang Abudourousuli, Suwen Chen, Yameng Hu, Wanying Qian, Xinyi Liao, Yingru Xu, Libing Song, Shuxia Zhang, Jun Li

**Affiliations:** ^1^ Key Laboratory of Liver Disease of Guangdong Province, The Third Affiliated Hospital, Sun Yat-sen University, Guangzhou, China; ^2^ Department of Biochemistry, Zhongshan School of Medicine, Sun Yat-sen University, Guangzhou, China; ^3^ State Key Laboratory of Oncology in South China, Cancer Center, Collaborative Innovation Center for Cancer Medicine, Sun Yat-sen University, Guangzhou, China; ^4^ Department of Oncology, The First Affiliated Hospital, Sun Yat-sen University, Guangzhou, China

**Keywords:** NKX2-8, breast cancer, bone metastasis, osteoclastogenesis, PTHrP

## Abstract

Bone metastasis is one of the most common distant metastasis of breast cancer, which could cause serious skeletal disease and increased cancer-related death. Therefore, identification of novel target(s) to develop therapeutics would improve patient outcomes. The role of NKX2-8 in modulation of bone remodeling was determined using osteoclastogenesis and micro-CT assays. The expression of NKX2-8 was examined *via* immunohistochemistry analysis in 344 breast cancer tissues. The mechanism underlying NKX2-8-mediated PTHrP downregulation was investigated using biotinylated deactivated Cas9 capture analysis, chromatin immunoprecipitation, co-immunoprecipitation assays. A bone-metastatic mouse model was used to examine the effect of NKX2-8 dysregulation on breast cancer bone metastasis and the impact of three PTHrP inhibitor on prevention of breast cancer bone metastasis. The downregulated expression of NKX2-8 was significantly correlated with breast cancer bone metastasis. *In vivo* bone-metastatic mouse model indicated that silencing NKX2-8 promoted, but overexpressing NKX2-8 inhibited, breast cancer osteolytic bone metastasis and osteoclastogenesis. Mechanistically, NKX2-8 directly interacted with HDAC1 on the PTHrP promoter, which resulted in a reduction of histone H3K27 acetylation, consequently transcriptionally downregulated PTHrP expression in breast cancer cells. Furthermore, targeting PTHrP effectively inhibited NKX2-8-downregulation-mediated breast cancer bone metastasis. Taken together, our results uncover a novel mechanism underlying NKX2-8 downregulation-mediated breast cancer bone metastasis and represent that the targeting PTHrP might be a tailored treatment for NKX2-8 silencing-induced breast cancer bone metastasis.

## Introduction

Recently, it has been recently reported that the incidence of breast cancer has become the highest and one of top leading cause of cancer death in the world (1), generally caused by the outgrowth of cancer cells in distant organs, such as bone, brain, liver and lungs (2). More than 70% advanced breast cancer develops bone metastasis that significantly reduced the quality of life and survival of patients (3, 4). However, current treatments for bone metastasis have limited efficacy. Despite the temporary effect of bisphosphonates and denosumab (an anti-receptor activator of NF-κB ligand (RANKL) monoclonal antibody) on decreasing the risk of skeletal-related events (SREs), no significant effect has been observed in terms of overall survival. Thus, it is an urgent necessity to identify the vital molecule(s) that contribute to bone metastasis of breast cancer, which could be used as diagnostic marker and novel treatment target for bone-metastasis of breast cancer.

Breast cancer bone metastasis are mostly osteolytic metastases, caused by recruitment and activation of osteoclasts to the tumor-bone interface to form aberrant bone resorption (5). Evidences proves that cancer-secreted factors directly or indirectly activated osteoclasts to absorb the bone matrix, leading to formation of a “bone pre-metastatic niche” to support cancer bone metastasis, thus generating a “vicious cycle” between bone-metastatic tumor cells and the bone pre-metastatic niche (4, 5). Parathyroid-hormone related peptide (PTHrP) is the most important osteoclast-activating factors released by cancer cells, including breast cancer cells (3). It has been proven that breast cancer cells do not express RANKL, but produce PTHrP to elevate RANKL secretion from osteoblasts to activate osteoclasts (6). Although it has been widely demonstrated that PTHrP plays the central role in breast cancer bone metastasis, the molecular mechanism in regulation of PTHrP expression remain largely unclear.

Human Nk2 homeobox 8 (NKX2-8), a NK2-related transcription factor, was reported to be downregulated in multiple tumors, which contributed to initiation, progression and development of cancer (7–13). Our previous studies have demonstrated that downregulation of NKX2-8 significantly contributed to malignant progression and development of bladder cancer (10) and esophageal squamous carcinoma (11), and deletion of NKX2-8 conferred chemoresistance on epithelial ovarian cancer (13). Herein, we demonstrated that NKX2-8 transcriptionally downregulated the PTHrP expression through directly interacting with histone deacetylase 1 (HDAC1) on the PTHrP promoter, resulting in a reduction of histone H3K27 acetylation, which transcriptionally downregulated PTHrP level in breast cancer. Silencing NKX2-8 induced PTHrP expression to activate osteoclasts, which generated a bone-metastasis supported “bone pre-metastatic niche”. These results not only demonstrated the crucial role of NKX2-8 reduction in osteoclastogenesis-induced breast cancer bone metastasis but also represent a potential therapeutic strategy to treat bone metastasis of NKX2-8-downregulated breast cancer.

## Materials and Methods

### Cell Lines and Cell Culture

The breast cancer cell lines, including MDA-MB-231 and SCP2, osteoclast precursors Raw 264.7 cells and osteoblast precursors MC3T3-E1 cells were cultured in Dulbecco’s modified Eagle’s medium (DMEM) (Gibco, Grand Island, NY, USA) plus with 10% fetal bovine serum (FBS; Gibco). SCP2 cell line was kindly provided by Prof. Guohong Hu in the School of Medicine, Chinese Academy of Sciences and Shanghai Jiao Tong University. The abovementioned cell lines have been examined the contamination of mycoplasma and authenticated by short tandem repeat (STR) fingerprinting at the department of Forensic in Sun Yat-Sen University (China).

### Plasmids, Retroviral Infection and Transfection

The retroviral vector pMSCV-neo was employed to construct the pMSCV-neo/NKX2-8 plasmid. Short hairpin RNAs (shRNAs) targeting NKX2-8, PTHrP, and HDAC1 were cloned into the pSuper Retro viral vector. The detailed information of shRNA oligonucleotides is presented in **Supplementary Table 1**. The region of human PTHrP promoter, which included nucleotides from −2000 to +500 around transcription start site, was subcloned into pGL3-Control luciferase reporter vector (Promega, Madison, WI, USA). Stable cell lines expressing NKX2-8, or NKX2-8 shRNA(s), PTHrP shRNA(s) and HDAC1 shRNA(s) were established by retroviral infection and 10 days selection with puromycin (0.5 µg/mL).

### RNA Extraction, Reverse Transcription and Real-Time PCR

The Trizol (Life Technologies, Carlsbad, CA, USA) reagent was used to extract total RNA from the indicated cells performed as the manufacturer’s instructions. The extracted RNA was reverse transcribed to cDNA, which was used in the quantitative real-time PCR step of the quantitative real-time reverse transcription PCR (qRT-PCR) protocol. The primers and probers used for qRT PCR were designed using the Primer Express v 2.0 software (Applied BioSystems, Foster City, CA, USA). The expression level of indicated genes was normalized to the expression of the housekeeping gene GAPDH (encoding glyceraldehyde-3-phosphate dehydrogenase) and calculated as 2^- [(Ct of gene) - (Ct of GAPDH)]^, which C_t_ indicated the cycle threshold for each gene. All primers are listed in **Supplementary Table 1**.

### Luciferase Assay

Total 1 × 10^3^ indicated cells were cultured in 48-well for 24 h and then transfected with control or correspond luciferase reporter plasmids (100 ng) and pRL-TK renilla plasmid (5ng) (Promega, Madison, WI) using the Lipofectamine 3000 reagent (Invitrogen, Carlsbad, CA, USA). The Dual Luciferase Reporter Assay Kit (Promega, Madison, WI) was used to measure the luciferase and renilla signals after transfection at 48h using the protocol provided by the manufacturer.

### Chromatin Immunoprecipitation (ChIP) Assay

The chromatin immunoprecipitation (ChIPs) assay kit (Cell Signaling Technology, Danvers, MA, USA) was used for ChIP assay according to protocol provided by the manufacturer’s instructions. In brief, the indicated cells were cultured on 100-mm culture dish around 70%~80% confluence and were fixed to cross-link proteins with DNA using 1% formaldehyde. The cell lysates were sonicated to shear DNA into small uniform fragments. Equal amounts of supernatants using anti-NKX2-8 (Abcam), or anti-HDAC1 (Abcam), or anti-H3K27ac (Abcam) and anti-IgG antibodies (Millipore, Billerica, MA) with protein G magnetic beads were immunoprecipitated overnight at 4°C. The cross-linked protein/DNA complexes were collected by magnetic pull down, and then were eluted from beads by elution buffer. PCR analysis with the indicated primers was conducted using the free DNA reversed from cross-linked protein/DNA complexes. The indicated ChIP primers are showed in **Supplementary Table 1**.

### Chemical Reagents

3 types of PTHrP inhibitors, the PTHrP neutralizing antibody (T-4512) were purchased from Bachem (Torrance, CA, USA). 6-thioguanine (6-TG) was purchased from Sigma-Aldrich (St. Louis, MO, USA), and PTHrP_7-34_ was purchased from GL Biochem (Shanghai, China).

### Enzyme-Linked Immunosorbent Assay (ELISA)

The PTHrP level in the culture medium from breast cancer cells was measured using a Human PTHrP (Parathyroid hormone-related protein) ELISA Kit (EH1058, FineTest, Wuhan Fine Biotech Co., Ltd., Wuhan, China), and analyzed according to the manufacturer’s instructions. The levels of transforming growth factor beta (TGF-β), RANKL, and osteoprotegerin (OPG) in culture medium were measured using mouse TGF-β ELISA Kit (ab119557), human RANKL ELISA Kit (ab213841), mouse RANKL ELISA Kit (ab100749), human OPG ELISA Kit (ab100617) and mouse OPG ELISA Kit (ab203365), respectively. The SpectraMax i3x Multi-Mode Microplate Reader (Molecular Devices, San Jose, CA, USA) was used to read data at 450 nm.

### Patient Information

A total of 20 tumor-adjacent normal breast tissues and 344 paraffin-embedded breast cancer samples were performed in this study. All samples that were histopathologically and clinically diagnosed at the third Affiliated Hospital, Sun Yat-sen Univeristy Cancer Center, and the First Affiliated Hospital from 2005 to 2019. The protocols used in this study were approved by the Institutional Research Ethics Committee of Sun Yat-sen University for the use of these clinical materials for research purposes. All Patients’ samples were obtained according to the Declaration of Helsinki and each patient signed a written informed consent for all the procedures.

### Immunohistochemistry (IHC) Assay

IHC assay was used to measure the NKX2-8 protein level *via* anti-NKX2-8 antibody (1:100; Sigma-Aldrich Cat# AV31856), and PTHrP protein level *via* anti-NKX2-8 antibody (1:100; LSBio Cat# LS-C31524-100) in 20 normal breast tissue and 344 breast cancer specimens, according previous report (12). Axio Imager.Z2 system (Carl Zeiss Co. Ltd., Jena, Germany) was used to capture the immunohistochemistry images. The degree of immunostaining of formalin-fixed, paraffin-embedded sections were reviewed and scored separately by two independent pathologists blinded to the histopathological features and patient data of the samples. The scores were determined by combining the proportion of positively-stained tumor cells and the intensity of staining. The scores given by the two independent pathologists were combined into a mean score for further comparative evaluation. Tumor cell proportions were scored as follows: 0, no positive tumor cells; 1, <10% positive tumor cells; 2, 10–35% positive tumor cells; 3, 35–75% positive tumor cells; 4, >75% positive tumor cells. The staining intensity was graded according to the following standard: 1, no staining; 2, weak staining (light yellow); 3, moderate staining (yellow brown); 4, strong staining (brown). The staining index (SI) was calculated as the product of the staining intensity score and the proportion of positive tumor cells. Using this method of assessment, we evaluated protein expression in normal breast tissues, breast cancer tissues and bone metastasis tissues by determining the SI, with possible scores of 0, 2, 3, 4, 6, 8, 9, 12, and 16. Samples with an SI ≥ 8 were determined as high expression and samples with an SI < 8 were determined as low expression. Cutoff values were determined on the basis of a measure of heterogeneity using the log-rank test with respect to overall survival.

### CAPTURE System

CAPTURE system using the biotinylated deactivated Cas9 (dCas9) was performed according to a previous report (14). In brief, the genomic locus-associated protein in the breast cancer cells transfected with the CAPTURE system, including a FB-dCas9 and a biotin ligase BirA (purchased from Addgene, Watertown, MA, USA; 100547 and 100548), and target-specific single guide RNAs (targeting the promoter of PTHrP, listed in **Supplementary Table 1**), were isolated using streptavidin purification and then for mass spectrometry analysis.

### Cell Growth Assay

A 3-(4, 5-Dimethylthiazol-2-yl)-2, 5-diphenyltetrazolium bromide (MTT) assay was employed to examine the cell growth. In brief, the indicated cells (8 × 10^3^) were cultured in 96-well plate at the indicated time point, and 0.5 mg/mL MTT was added to each well for 4 h. Then removing MTT, adding dimethyl sulfoxide (DMSO), and mixing vigorously. The SpectraMax i3x Multi-Mode Microplate Reader (Molecular Devices) was used to measure the absorbance at 490nm.

### Osteoclastogenesis Assay

Osteoclast precursor cells (1 × 10^5^) were cultured on 24-well clusters containing glass coverslips (Thermo Fisher Scientific, Waltham, MA, USA) and grown in the conditioned media (CM), alone or treated with anti-PTHrP antibody (10 μg/mL), or 6-TG (10 μM), or PTHrP_7-34_ (0.5 μM). Media were changed at every other day. Osteoclasts were counted on day 6. The osteoclasts cultured on plastic dishes were fixed with 4% paraformaldehyde/phosphate-buffered saline (PBS) and tartrate-resistant acid phosphatase (TRAP) in the cells was stained using a commercial kit (387A-1KT; Sigma-Aldrich). The TRAP-positive multinucleated cells that contain > 3 nuclei defined as osteoclasts.

### Xenografted Tumor Models

The animal study was reviewed and approved by the Sun Yat-sen University Animal Care Committee. Bone-metastasis assay was performed using the indicated luciferase-expressing breast cancer cells (1×10^5^) that were intracardially injected into nu/nu nude mice (5 weeks old). Anti-PTHrP antibody (Abcam) or anti-rabbit IgG were administered intraperitoneally twice a week. The 6-TG (1.0 mg/kg in 100 μL of PBS), or PTHrP_7-34_ (200 μg/kg in μL of PBS) was injected subcutaneously daily. The SIEMENS micro-computed tomography (μCT) system (SIEMENS, Munich, Germany) was used to detect the osteolytic lesions in tibia and femur of hind limb. The bones of mice were harvested for further analysis.

### *In Vivo* Quantification of Osteoclasts

Hind limbs were fixed in paraformaldehyde solution (4%), decalcified in 14.3% EDTA for 4 days at 37°C with daily changes of EDTA, and then embedded in paraffin wax. Sections were stained with hematoxylin and eosin using Mayer’s hematoxylin solution, stained with TRAP (using a TRAP kit, 387A-1KT; Sigma-Aldrich) according to the manufacturer’s protocols. The numbers of TRAP^+^ osteoclasts were determined on a 3 mm length of endocortical surface and viewed under an optical microscope (Olympus, DP72, Tokyo, Japan).

### Statistics

All the data presented in this study were showed as the mean ± standard deviation (SD) and n represents the number of independent experiments performed on different mice, or different batches of cells, or different clinical tissues. Statistical analysis was performed either the Student’s two-tailed t-test or one-way analysis of variance (ANOVA). Bivariate correlations were calculated between study variables using Spearman’s rank correlation coefficients. Survival curves were plotted using the Kaplan-Meier method was used to plot survival curves that were compared using the log-rank test. Univariate and multivariate Cox regression analyses were used to analyze the significance of various variables for survival. The P-values that less than were considered statistically significant. The GraphPad Prism 7 (GraphPad Inc., La Jolla, CA, USA) and SPSS 19.0 (IBM Corp., Armonk, NY, USA) statistical software were used for statistical analysis and P-values were represented as *P < 0.05, **P < 0.01, ***P < 0.001, and N.S. means not significant (P > 0.05).

## Results

### Reduced NKX2-8 Is Associated With Progression of Bone-Metastasis in Breast Cancer

**Figure 1:**
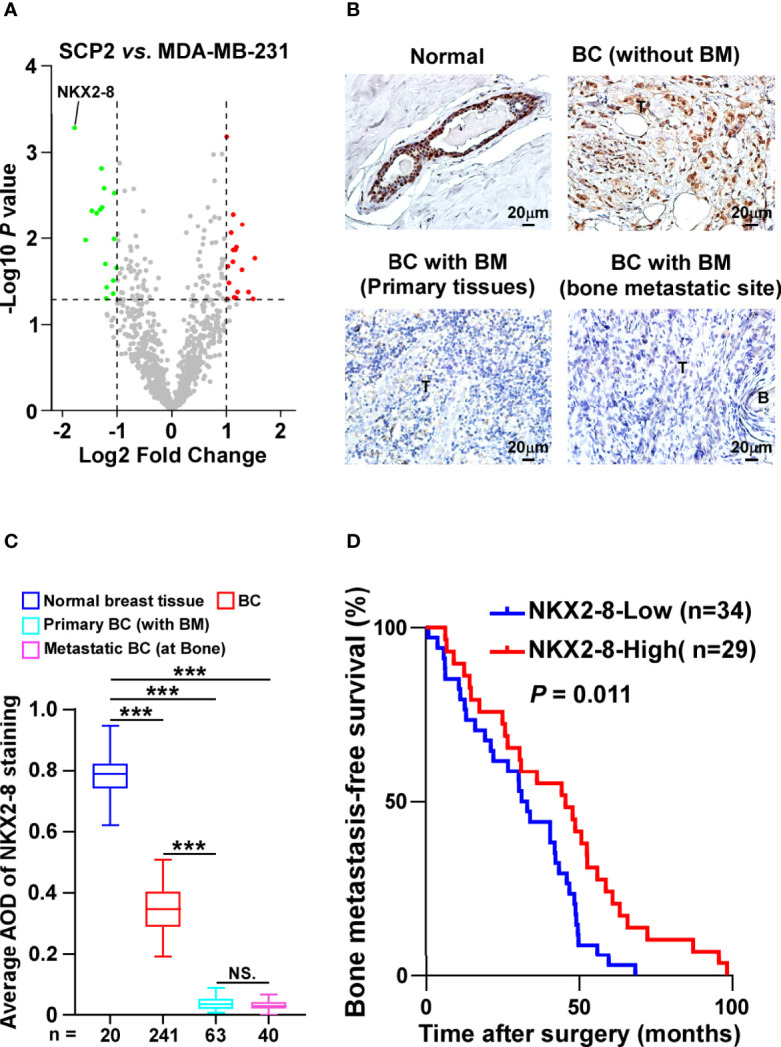
NKX2-8 is associated with progression of bone-metastasis in breast cancer. **(A)** The upregulated and downregulated proteins in SCP2 cells compared with MDA-MB-231 cells were analyzed using Volcano plot. **(B, C)** Representative IHC images **(B)** and quantification **(C)** of NKX2-8 level in normal breast tissue (n = 20), primary non-bone metastatic breast cancer tissues (n = 241), primary bone-metastatic breast cancer tissues (n = 63), and bone-metastatic breast cancer tissues (n = 40). Scale bar, 50 µm. **(D)** Kaplan-Meier analysis of bone metastasis-free survival curves in patients with NKX2-8 high- *vs.* low-expressed breast cancer with bone metastasis (n = 63; *P* = 0.011, log-rank test). *** means *P* < 0.001, N.S. means not significant (P > 0.05).

SCP2 cell line, which is the bone-tropism cell line derived from MDA-MB-231 cells, is used to study the bone-tropism of breast cancer metastasis (15). To screen the key factors involved in breast cancer bone metastasis, MS-based proteomics was performed in SPC2 and MDA-MB-231-parental cells. Analysis of protein profiling showed that a total of 34 proteins, including 18 elevated proteins and 16 reduced proteins, were dysregulated in SPC2 cells compared with MDA-MB-231-parental cells (**Figure 1A** and **Supplementary Table 2**). Among them, NKX2-8 levels were found to be significantly decreased in bone-metastatic tissues compared to normal breast tissues, or non-metastatic breast cancer tissues, respectively (**Figures 1B, C**, and **Supplementary Figure 1**). Furthermore, statistical analysis showed that patients with NKX2-8 high-expressing breast cancer had much longer bone-metastasis-free survival than the patients with NKX2-8 low-expressing breast cancer (*P* = 0.011 and **Figure 1D**). Taken together, our results indicate that the reduced NKX2-8 is linked to the development of bone-metastasis in breast cancer.

### NKX2-8 Suppresses Bone Metastasis of Breast Cancer Cells

**Figure 2:**
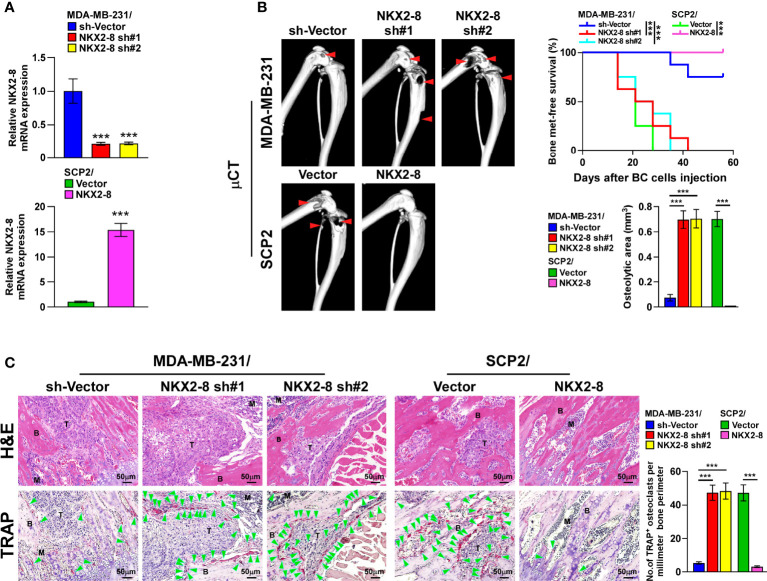
NKX2-8 silencing promotes bone metastasis in breast cancer. **(A)** Real-time PCR analysis of NKX2-8 expression in sh-vector- and NKX2-8-shRNA(s)-transduced SCP2 cells and in vector- and NKX2-8-transduced MDA-MB-231 cells. GAPDH served as the loading control. **(B)** Left: µCT images of bone lesions from representative mice. Right: Quantification of the μCT osteolytic lesion area, and Kaplan-Meier bone metastasis-free survival curve of the indicated mice groups in experimental metastasis phase (n = 8/group). **(C)** Histological H&E images (left-upper) and TRAP images (left-lower), and quantification (right) of the osteolytic area and TRAP-positive osteoclasts as shown in the indicated mice (n = 8/group). Scale bar, 50 µm. BC, Breast Cancer; BM, Bone Metastasis. * means *P* < 0.05, *** means *P* < 0.001.

The biological role of NKX2-8 in breast cancer organ-specific metastasis was then examined using NKX2-8-silenced SCP-2 and NKX2-8–overexpressing MDA-MB-231 breast cancer cell lines (**Figure 2A**), and then injected intracardially the corresponding cells into nude mice. The NKX2-8-silenced breast cancer cells-injected mice exhibited earlier bone metastases, as revealed by μCT analysis, and histology examination (**Figures 2B, C**). Consistently, compared with that in the control mice, the NKX2-8-overexpressing breast cancer cells displayed delayed bone metastases, and reduced bone metastasis lesions/osteolytic areas (**Figures 2B, C**). Histological TRAP staining showed that NKX2-8-overexpressing breast cancer cells significantly suppressed activation of osteoclasts (**Figure 2C**). Collectively, these results demonstrated the NKX2-8 specifically inhibits bone metastasis of breast cancer cells by reducing osteoclastogenesis.

### Downregulation of NKX2-8-Induced PTHrP Promotes Osteoclastogenesis

**Figure 3:**
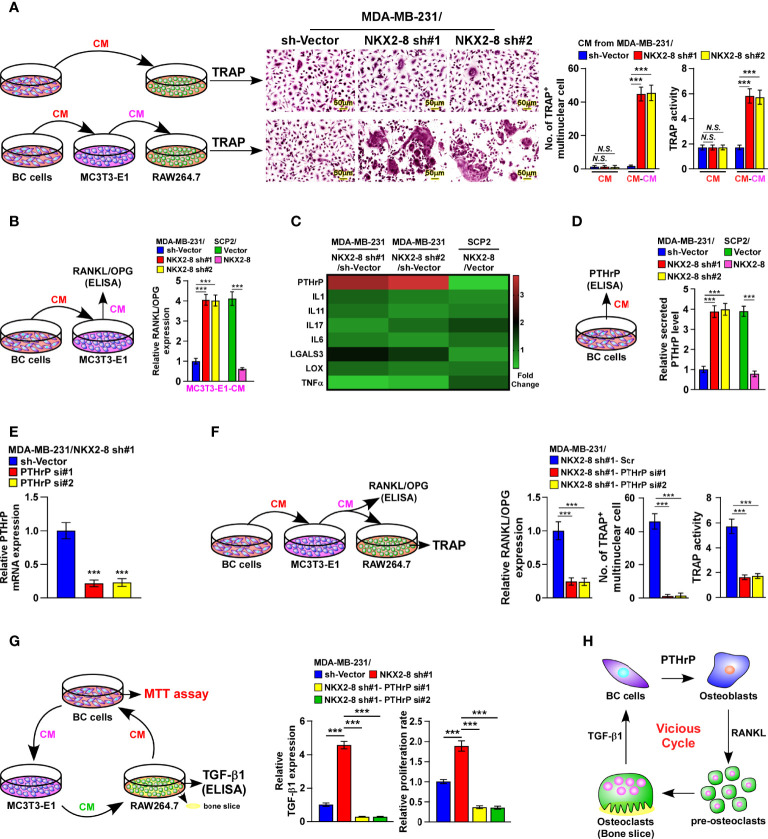
NKX2-8 silencing-induced PTHrP promotes osteoclastogenesis *in vitro*. **(A)** Left: schematic illustration of osteoclastogenesis under treatment with CM from breast cancer cells or from osteoblasts pretreated with CM from breast cancer cells. Middle: osteoclast differentiation assays assessed using TRAP staining cultured CM obtained from the indicated cells. Right: Quantification of the number of TRAP-positive multinuclear osteoclasts and TRAP activity. **(B)** ELISA analysis of the RANKL/OPG ratio in CM from osteoblast precursor cells MC3T3-E1 cells cultured in the CM obtained from the indicated breast cancer cells. **(C)** Real-time PCR analysis indicating osteoclastogenesis regulator expression in the indicated cells. The pseudocolor represents the intensity scale of genes in the indicated cells generated by a log2 transformation. GAPDH serves as the loading control. **(D)** ELISA analysis of secreted PTHrP levels in CM from the indicated cells. **(E)** Real-time PCR analysis of PTHrP levels in sh-vector- and PTHrP-shRNA(s)-transduced MDA-MB-231/NKX2-8 sh#1 cells. GAPDH served as the loading control. **(F)** Left: schematic illustration of osteoclastogenesis under treatment with CM from osteoblasts pretreated with CM from breast cancer cells. Right: ELISA analysis of the RANKL/OPG ratio in CM from osteoblast precursor cells MC3T3-E1 cells in the presence of CM from the indicated breast cancer cells, and quantification of the TRAP-positive multinuclear osteoclasts number and TRAP activity cultured in the CM obtained from the indicated cells. **(G)** Left: schematic illustration of breast cancer CM-induced “vicious cycle” between the indicated cells. Middle: The TGF-β1 levels analyzed using ELISA assay in CM from RAW 264.7 cells cultured onto the bone slice in CM obtained from the breast cancer cells. Right: MTT assay analysis of proliferation rate of indicated cells from experiment in left panel. **(H)** Schematic illustration of PTHrP-induced “vicious cycle” between the indicated cells. BC: Breast Cancer. N.S. means not significant (P > 0.05), *** means *P* < 0.001.

Interestingly, similar with treatment with the conditioned media (CM) from control cells, there was no effect on osteoclastogenesis when osteoclasts were treated directly with that of NKX2-8-silenced cells (**Figure 3A**). Nevertheless, CM from osteoblasts was found to significantly increase the TRAP-positive multinuclear osteoclasts number and enhance the TRAP enzymatic activity, when osteoblasts were pretreated with CM from NKX2-8-silenced cells (**Figure 3A**). There results suggested that CM from NKX2-8-silenced cells indirectly promotes osteoclastogenesis. Considering that the RANKL/OPG axis is vital for osteoclastogenesis, we next examined the RANKL/OPG ratio in osteoblasts under induction by CM from breast cancer cells. The relative RANKL/OPG ratio was dramatically elevated after treatment with CM from NKX2-8-silenced cells, but was decreased after treatment with CM from NKX2-8-overexpressing cells (**Figure 3B**). Consistently, the expression of differentiation marker and activation marker of osteoclasts, such as Acp5, Ctsk, Nfat-c1, C-fos, and Dc-stamp, were significantly upregulated in osteoclasts treated with CM from osteoblasts that pretreated with CM from NKX2-8-silenced breast cancer cells, but decreased in response to overexpression of NKX2-8 (**Supplementary Figure 2**). These results indicated that the NKX2-8 silencing induced-secretome promotes osteoclastogenesis *via* osteoblasts-secreted RANKL.

Among the osteoclastogenesis regulators that have been reported previously (16–19), PTHrP was one of the most upregulated secreted proteins in NKX2-8-silenced cells, but was downregulated in NKX2-8-overexpressing cells (**Figure 3C**). Consistently, the secreted PTHrP protein levels were also drastically decreased in NKX2-8-overexpressed cells, but elevated in NKX2-8-silenced cells, (**Figure 3D**), suggesting the potential role of PTHrP in breast cancer bone-metastasis.

Silencing PTHrP abrogated NKX2-8-silenced induction of RANKL secretion, the TRAP-positive multinuclear osteoclasts number, the enzymatic activity of TRAP (**Figures 3E, F**), which demonstrated that NKX2-8 silencing induced-PTHrP promotes osteoclastogenesis *via* osteoblasts-secreted RANKL. Moreover, the NKX2-8 silencing-promoted vicious cycle was significantly blocked by silencing PTHrP, as indicated by downregulation of bone matrix-released TGF-β and reduced growth rates of breast cancer cells (**Figure 3G**). Taken together, our results strongly indicated a critical role of the NKX2-8/PTHrP/RANKL axis in the regulation of osteoclastogenesis *in vivo*, which resulted in a vicious cycle between tumor cells and osteoclasts (**Figure 3H**).

### NKX2-8 Transcriptionally Represses the Expression of PTHrP

**Figure 4:**
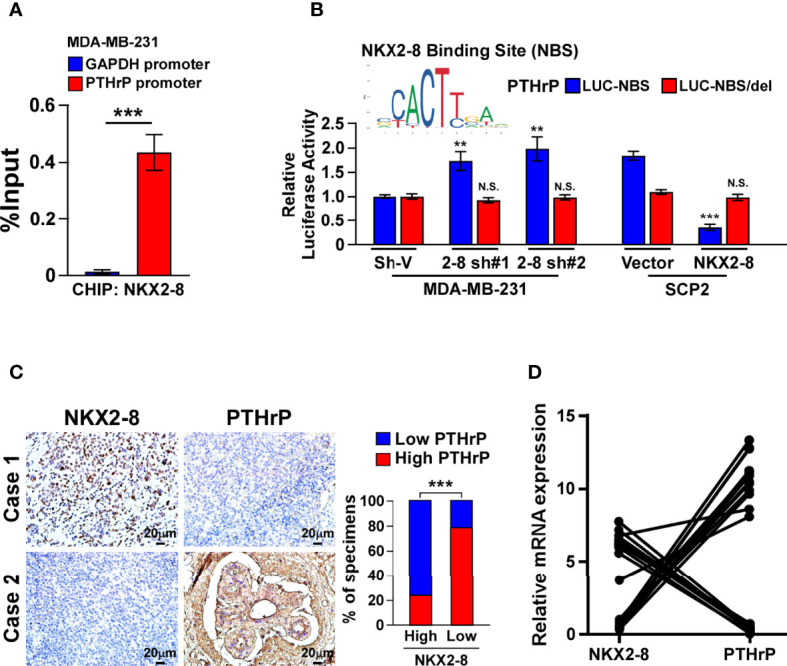
NKX2-8 transcriptionally represses PTHrP. **(A)** ChIP analysis of the enrichment of NKX2-8 on promoter of PTHrP gene in MDA-MB-231 cells. **(B)** Relative luciferase activities of the PTHrP promoter in the indicated breast cancer cells. **(C)** NKX2-8 levels were negatively associated with PTHrP expression in breast cancer tissues (n = 304). The representative IHC staining images (left) and expression correlation (right) of NKX2-8 and PTHrP protein in breast cancer tissues. Scale bars, 20 µm. **(D)** Relative expression of NKX2-8 and PTHrP in primary breast cancer tissues (n = 20) with bone metastasis (n = 10; P < 0.001). ** means *P* < 0.01., *** means *P* < 0.001 N.S. means not significant (P > 0.05).

Next, the role of NKX2-8 reduction in PTHrP expression were examined. As shown in **Figure 4A**, the NKX2-8 ChIP assay results suggested that NKX2-8 was most significantly associated with the PTHrP promoter in MDA-MB-231 cells. Furthermore, the NKX2-8-silenced cells displayed increased, but NKX2-8-overexpressing cell exhibited decreased, the luciferase activities of genes with a NKX2-8-specific binding site (NBS) (**Figure 4B**). Whereas, we did not observe the effect on the luciferase activities of the NKX2-8 NBS-deleted promoter (**Figure 4B**). The reverse correlation adverse of NKX2-8 with PTHrP expression was also demonstrated using IHC analysis in breast cancer tissues, which NKX2-8 expression was adversely associated with the PTHrP level (**Figure 4C**). Importantly, no correlation of NKX2-8 and PTHrP was found in the breast cancer tissues with other organs metastasis (**Supplementary Figure 3**). Real-time PCR analysis revealed that the PTHrP expression was downregulated in the NKX2-8 high-expressing breast but upregulated in the NKX2-8 low-expressing (**Figure 4D**). Therefore, these results provided further evidence that NKX2-8 transcriptional downregulates PTHrP expression *via* directly targeting on its promoters.

### The NKX2-8/HDAC1 Complex Is Involved in NKX2-8-Inhibited PTHrP Expression

**Figure 5:**
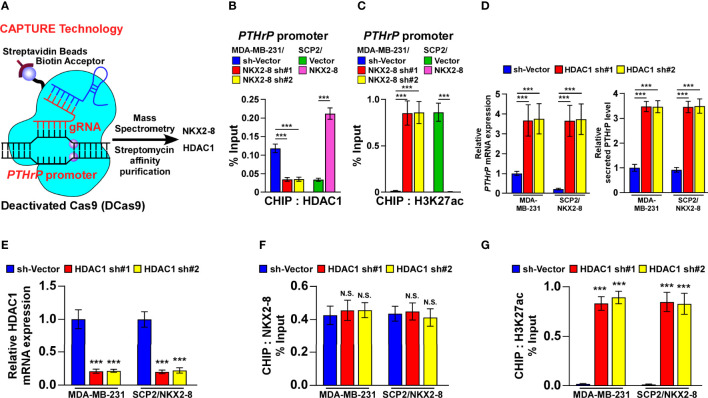
NKX2-8 interacts with HDAC1 in NKX2-8-inhibited PTHrP transcription. **(A)** Schematic diagram of interaction between NKX2-8 and the HDAC1 in the PTHrP promoter using CAPATURE approach. **(B)** ChIP analysis of the enrichment of HDAC1 in the PTHrP promoter in the indicated breast cancer cells. **(C)** ChIP analysis of the enrichment of H3K27ac in the PTHrP promoter in the indicated breast cancer cells. **(D)** Real-time PCR analysis of PTHrP mRNA (left) and ELISA analysis of serum PTHrP (right) expression in the indicated cells. GAPDH was used as the loading control. **(E)** The relative expression of HDAC1 in the indicated cells using real-time PCR analysis. GAPDH served as the loading control. **(F)** ChIP analysis of the association of NKX2-8 with the promoter of PTHrP gene in the indicated breast cancer cells. **(G)** The relative enrichment of H3K27ac on the PTHrP promoter determined by ChIP analysis in the indicated breast cancer cells. N.S. means not significant (P > 0.05), *** means *P* < 0.001.

In order to investigate the molecular mechanism in which NKX2-8-mediated transcriptional suppression of PTHrP, we performed the biotinylated deactivated Cas9 (dCas9) capture analysis and found that both NKX2-8 and HDAC1 were identified to target on the PTHrP promoter in MDA-MB-231 cells (**Figure 5A** and **Supplementary Table 3**). Furthermore, ChIP assays revealed that downregulation of NKX2-8 significantly reduced, whereas upregulation of NKX2-8 enriched, the HDAC1 level on the PTHrP promoter in breast cancer cells (**Figure 5B**). Conversely, silencing NKX2-8 drastically increased, but overexpressing NKX2-8 decreased, the enrichment of H3K27 acetylation (H3K27ac) on the promoter of PTHrP gene in breast cancer cells (**Figure 5C**), which demonstrated that NKX2-8 inhibited PTHrP expression *via* HDAC1 to reduce H3K27ac on the promoter of PTHrP. Meanwhile, silencing HDAC1 significantly increased the PTHrP expression in NKX2-8 overexpression cells (**Figure 5D**). These results suggested that HDAC1 played an important role in NKX2-8-mediated PTHrP expression. In line with this hypothesis, we found that knockdown of HDAC1 has no impact on the enrichment of NKX2-8, but significantly increased the level of H3K27ac, on the PTHrP promoter (**Figures 5E–G**), which further supported the notion that NKX2-8 inhibits PTHrP expression *via* HDAC1 to reduce H3K27ac on the promoter of PTHrP.

**Figure 6:**
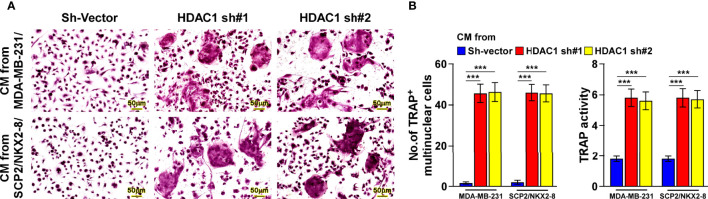
NKX2-8 represses osteoclastogenesis *via* HDAC1. **(A)** TRAP staining images of the indicated CM-treated pre-osteoclasts. Scale bar, 20μm or 50μm. **(B)** The number quantification of TRAP-positive multinuclear cells and examination of TRAP activity. *** means *P* < 0.001.

Consistently, silencing HDAC1 in the breast cancer cells significantly abolished the reductive effect of NKX2-8 on the formation of TRAP^+^-multinuclear osteoclasts and TRAP enzymatic activity (**Figures 6A, B**). Taken together, these results suggest that the NKX2-8/HDAC1 repressor complex is involved in NKX2-8-inhibited PTHrP expression in breast cancer cells.

### Targeting PTHrP Inhibit NKX2-8 Silencing-Induced Osteoclastogenesis and Bone Metastasis

**Figure 7:**
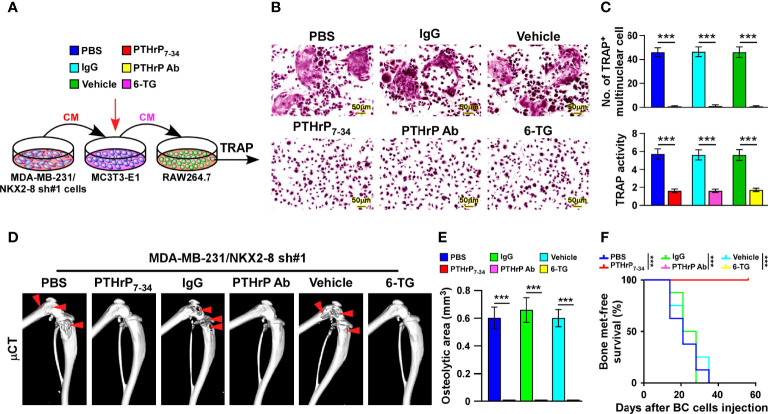
Targeting PTHrP inhibits NKX2-8 silencing-induced bone metastasis. **(A)** Schematic illustration of osteoclastogenesis in the indicated condition. **(B)** Osteoclast differentiation assays analyzed as shown by TRAP staining of the indicated CM-treated pre-osteoclasts with addition of PBS or PTHrP_7-34_, IgG or anti-PTHrP antibody, or vehicle or 6-TG. **(C)** Quantification of the number of TRAP-positive multinuclear cells (upper) and examination of TRAP activity (lower). **(D)** µCT images of bone lesions from mice injected with MDA-MB-231/NKX2-8 sh#1, and treated with PBS or PTHrP_7-34_ or IgG or anti-PTHrP antibody or vehicle or 6-TG. **(E, F)** Normalized BLI signals of bone metastases quantification of the μCT osteolytic lesion area **(E)**, and Kaplan-Meier bone metastasis-free survival curve **(F)** of the indicated mice in the experimental metastasis phase (n = 8/group). *** means *P* < 0.001.

To determine the critical effect of tumor-derived PTHrP on NKX2-8 silencing-mediated osteoclastogenesis, the osteoclast precursor cells was treated with three types of PTHrP inhibitors, including the peptide antagonist PTHrP_7–34_, the neutralizing antibody and the chemical inhibitor 6-thioguanine (6-TG), under conditions of CM from NKX2-8-silenced cells. Osteoclastogenesis analyses indicated that all three type of inhibitors efficiently impaired the osteoclastogenesis effects of the CM from NKX2-8-silenced cells, as PTHrP inhibition completely abolishing the induced effects of NKX2-8 silencing on the TRAP-positive multinuclear osteoclasts and TRAP enzymatic activity (**Figures 7A–C**). These results further supported the notion that NKX2-8 inhibited osteoclastogenesis *via* PTHrP *in vitro*. Moreover, we found that treatment with all three PTHrP inhibitor exhibited dramatic blocked effect on vicious cycle induced by silencing NKX2-8, as indicated by decreased level of bone matrix-released TGF-β1 and slower growth rates of breast cancer cells (**Supplementary Figure 4**). Therefore, our results indicate that targeting PTHrP contributes to inhibition of the osteoclastogenesis and vicious cycle induced by NKX2-8 silencing.

We injected NKX2-8 silenced MDA-MB-231 cells intracardially into nude mice and treated them with PTHrP_7–34_ or anti-PTHrP antibody or 6-TG, which markedly suppressed the bone metastasis signals and bone destruction, as monitored by μCT analysis (**Figure 7D**). Consistently, compared with that in the control mice, the mice treated with PTHrP_7–34_ or anti-PTHrP antibody or 6-TG displayed no bone metastases and bone metastasis lesions/osteolytic areas (**Figures 7E, F**). Collectively, our data demonstrate that targeting PTHrP inhibited NKX2-8 silencing-induced osteoclastogenesis and bone metastasis.

## Discussion

As the most prevalent form of metastasis, bone metastasis occurs over 70% breast cancer patients with advanced diseases (20). It can cause bone pain, osteoporosis, pathological fractures, and other skeletal-related events, which significantly reduce the patient’s quality of life, and can even be lethal (4, 21). The outcome of breast cancer with bone metastasis is extremely poor, which a 1-year survival rate of breast cancer patient is only 40-59% (22). Although current therapeutic modalities, including chemoradiotherapy and anti-osteolytic drugs, have made significant improvement on reduce the incidence rate associated with bone metastasis, these treatments only provide minimal benefit to patients` survival (4). Therefore, developing novel effective therapeutic strategies to treat patients with bone metastasis of breast cancer is urgent. In this study, we observed that NKX2-8-silencing-induced PTHrP protein plays a vital role in bone metastasis of breast cancer by promoting osteoclastogenesis. Importantly, targeting PTHrP significantly decreased osteoclastogenesis and effectively suppressed the progression of breast cancer bone metastasis. Therefore, these findings shed light on a potential mechanism underlying breast cancer bone metastasis and might represent a potential clinical strategy for treatment of breast cancer bone metastasis.

Breast cancer cells have the ability of “organotropic metastasis”, i.e., preferential metastasis to specific organs, which is regulated by subtypes of breast cancer, in which the tumor-induced pre-metastatic niche plays a vital role in engrafting and surviving of metastatic cells (23). It has been reported that only a very small fraction of the breast cancer cells possessing the ability to form highly aggressive, osteolytic bone metastases. Thus, we used SCP2 cells and MDA-MB-231, a derivative bone-tropism cell line that was isolated by Yibin Kang et al. (15). We found that NKX2-8 levels were markedly decreased in SCP2 cells compared with those in MDA-MB-231 parental cells. Subsequent experiments revealed an organ-specific correlation between NKX2-8 expression and bone metastasis. Importantly, our findings demonstrated that silencing NKX2-8 in breast cancer cells influenced their ability to affect the pre-metastatic niche. Silencing of NKX2-8 in breast cancer cells activated PTHrP transcription. Consequently, PTHrP derived from NKX2-8 silenced-breast cancer cells contribute to formation of pre-bone metastatic niche through alteration of RANKL/OPG ration *via* acting on osteoblasts, resulting in instigating osteoclastogenesis that led to metastatic bone destruction.

NKX2-8 is a transcription factor that participates in the progression or chemoresistance in multiple tumors (7–13). Our results showed that NKX2-8 plays a critical role in breast cancer bone metastasis. Specifically, NKX2-8 interacts with HDAC1 to form a complex that suppresses PTHrP transcription in breast cancer cells with no bone metastasis, while silencing NKX2-8 could eliminate its inhibition of bone metastasis. Our results provided a new paradigm for NKX2-8 in cancer, especially in bone metastasis, in which it was involved in reshaping the bone microenvironment for and enhance metastasis.

Overall, the present data provided in current study suggest a potential therapeutic application of PTHrP inhibitors for treatment and prevention of downregulation of NKX2-8-induced breast cancer bone metastasis.

## Data Availability Statement

The original contributions presented in the study are included in the article/**Supplementary Material**. Further inquiries can be directed to the corresponding authors.

## Ethics Statement

The animal study was reviewed and approved by Sun Yat-sen University Animal Care Committee.

## Author Contributions

AA designed the experiments and analyzed data. YH performed *in vitro* cell studies. SC performed the xenograft tumor experiments. WQ performed staining, immunohistochemical and pathological analysis. XL performed the CHIP, real time PCR, immunoprecipitation and western blot. YX analyzed mass spectrometry data. LS, SZ, and JL supervised the whole study and wrote the paper. All authors contributed to the article and approved the submitted version.

## Funding

This work was supported by National Natural Science Foundation of China (No. 82030078, 81830082, 82072609, 81621004 and 82003128).

## Conflict of Interest

The authors declare that the research was conducted in the absence of any commercial or financial relationships that could be construed as a potential conflict of interest.

## Publisher’s Note

All claims expressed in this article are solely those of the authors and do not necessarily represent those of their affiliated organizations, or those of the publisher, the editors and the reviewers. Any product that may be evaluated in this article, or claim that may be made by its manufacturer, is not guaranteed or endorsed by the publisher.

## References

[1] Sung H Ferlay J Siegel RL Laversanne M Soerjomataram I Jemal A. Global Cancer Statistics 2020: Globocan Estimates of Incidence and Mortality Worldwide for 36 Cancers in 185 Countries. CA Cancer J Clin (2021) 71(3):209–49. doi: 10.3322/caac.21660 33538338

[2] Hess KR Varadhachary GR Taylor SH Wei W Raber MN Lenzi R. Metastatic Patterns in Adenocarcinoma. Cancer (2006) 106(7):1624–33. doi: 10.1002/cncr.21778 16518827

[3] Mundy GR. Metastasis to Bone: Causes, Consequences and Therapeutic Opportunities. Nat Rev Cancer (2002) 2(8):584–93. doi: 10.1038/nrc867 12154351

[4] Weilbaecher KN Guise TA McCauley LK. Cancer to Bone: A Fatal Attraction. Nat Rev Cancer (2011) 11(6):411–25. doi: 10.1038/nrc3055 PMC3666847 21593787

[5] Ell B Kang Y. Snapshot: Bone Metastasis. Cell (2012) 151(3):690–e1. doi: 10.1016/j.cell.2012.10.005 23101634

[6] Thomas RJ Guise TA Yin JJ Elliott J Horwood NJ Martin TJ. Breast Cancer Cells Interact With Osteoblasts to Support Osteoclast Formation. Endocrinology (1999) 140(10):4451–8. doi: 10.1210/endo.140.10.7037 10499498

[7] Kajiyama Y Tian J Locker J. Regulation of Alpha-Fetoprotein Expression by Nkx2.8. Mol Cell Biol (2002) 22(17):6122–30. doi: 10.1128/MCB.22.17.6122-6130.2002 PMC134004 12167706

[8] Hagihara A Miyamoto K Furuta J Hiraoka N Wakazono K Seki S. Identification of 27 5' Cpg Islands Aberrantly Methylated and 13 Genes Silenced in Human Pancreatic Cancers. Oncogene (2004) 23(53):8705–10. doi: 10.1038/sj.onc.1207783 15467763

[9] Harris T Pan Q Sironi J Lutz D Tian J Sapkar J. Both Gene Amplification and Allelic Loss Occur at 14q13.3 in Lung Cancer. Clin Cancer Res (2011) 17(4):690–9. doi: 10.1158/1078-0432.CCR-10-1892 PMC3041868 21148747

[10] Yu C Zhang Z Liao W Zhao X Liu L Wu Y. The Tumor-Suppressor Gene Nkx2.8 Suppresses Bladder Cancer Proliferation Through Upregulation of Foxo3a and Inhibition of the Mek/Erk Signaling Pathway. Carcinogenesis (2012) 33(3):678–86. doi: 10.1093/carcin/bgr321 22223847

[11] Lin C Song L Gong H Liu A Lin X Wu J. Nkx2-8 Downregulation Promotes Angiogenesis and Activates Nf-Kappab in Esophageal Cancer. Cancer Res (2013) 73(12):3638–48. doi: 10.1158/0008-5472.CAN-12-4028 23604637

[12] Qu L Deng B Zeng Y Cao Z. Decreased Expression of the Nkx2.8 Gene Correlates With Tumor Progression and a Poor Prognosis in Hcc Cancer. Cancer Cell Int (2014) 14:28. doi: 10.1186/1475-2867-14-28 24678995 PMC4011771

[13] Zhu J Wu G Song L Cao L Tan Z Tang M. Nkx2-8 Deletion-Induced Reprogramming of Fatty Acid Metabolism Confers Chemoresistance in Epithelial Ovarian Cancer. EBioMedicine (2019) 43:238–52. doi: 10.1016/j.ebiom.2019.04.041 PMC6562195 31047858

[14] Liu X Zhang Y Chen Y Li M Zhou F Li K. *In Situ* Capture of Chromatin Interactions by Biotinylated Dcas9. Cell (2017) 170(5):1028–43.e19. doi: 10.1016/j.cell.2017.08.003 28841410 PMC6857456

[15] Kang Y Siegel PM Shu W Drobnjak M Kakonen SM Cordon-Cardo C. A Multigenic Program Mediating Breast Cancer Metastasis to Bone. Cancer Cell (2003) 3(6):537–49. doi: 10.1016/s1535-6108(03)00132-6 12842083

[16] Jones DH Kong YY Penninger JM. Role of Rankl and Rank in Bone Loss and Arthritis. Ann Rheum Dis (2002) 61 (Suppl 2):ii32–9. doi: 10.1136/ard.61.suppl_2.ii32 PMC1766717 12379618

[17] Zhang S Xu Y Xie C Ren L Wu G Yang M. Rnf219/Alpha-Catenin/Lgals3 Axis Promotes Hepatocellular Carcinoma Bone Metastasis and Associated Skeletal Complications. Adv Sci (Weinh) (2021) 8(4):2001961. doi: 10.1002/advs.202001961 33643786 PMC7887580

[18] Nakajima K Kho DH Yanagawa T Harazono Y Hogan V Chen W. Galectin-3 Cleavage Alters Bone Remodeling: Different Outcomes in Breast and Prostate Cancer Skeletal Metastasis. Cancer Res (2016) 76(6):1391–402. doi: 10.1158/0008-5472.CAN-15-1793 PMC4863655 26837763

[19] Cox TR Rumney RMH Schoof EM Perryman L Hoye AM Agrawal A. The Hypoxic Cancer Secretome Induces Pre-Metastatic Bone Lesions Through Lysyl Oxidase. Nature (2015) 522(7554):106–10. doi: 10.1038/nature14492 PMC4961239 26017313

[20] Coleman RE. Metastatic Bone Disease: Clinical Features, Pathophysiology and Treatment Strategies. Cancer Treat Rev (2001) 27(3):165–76. doi: 10.1053/ctrv.2000.0210 11417967

[21] Coleman RE. Clinical Features of Metastatic Bone Disease and Risk of Skeletal Morbidity. Clin Cancer Res (2006) 12(20 Pt 2):6243s–9s. doi: 10.1158/1078-0432.CCR-06-0931 17062708

[22] Cetin K Christiansen CF Svaerke C Jacobsen JB Sorensen HT. Survival in Patients With Breast Cancer With Bone Metastasis: A Danish Population-Based Cohort Study on the Prognostic Impact of Initial Stage of Disease at Breast Cancer Diagnosis and Length of the Bone Metastasis-Free Interval. BMJ Open (2015) 5(4):e007702. doi: 10.1136/bmjopen-2015-007702 PMC4420974 25926150

[23] Chen W Hoffmann AD Liu H Liu X. Organotropism: New Insights Into Molecular Mechanisms of Breast Cancer Metastasis. NPJ Precis Oncol (2018) 2(1):4. doi: 10.1038/s41698-018-0047-0 29872722 PMC5871901

