# Supplementary material for: NKX2-8/PTHrP Axis-Mediated Osteoclastogenesis and Bone Metastasis in Breast Cancer
Source: Front Oncol. 2022 May 30;12:907000. doi: 10.3389/fonc.2022.907000 (PMC9189290; doi:10.3389/fonc.2022.907000)
Supplement: Supplementary file 1 [file DataSheet_1.docx]

Supplementary Material

# Supplementary Figures and Tables

## Supplementary Figures and Legends


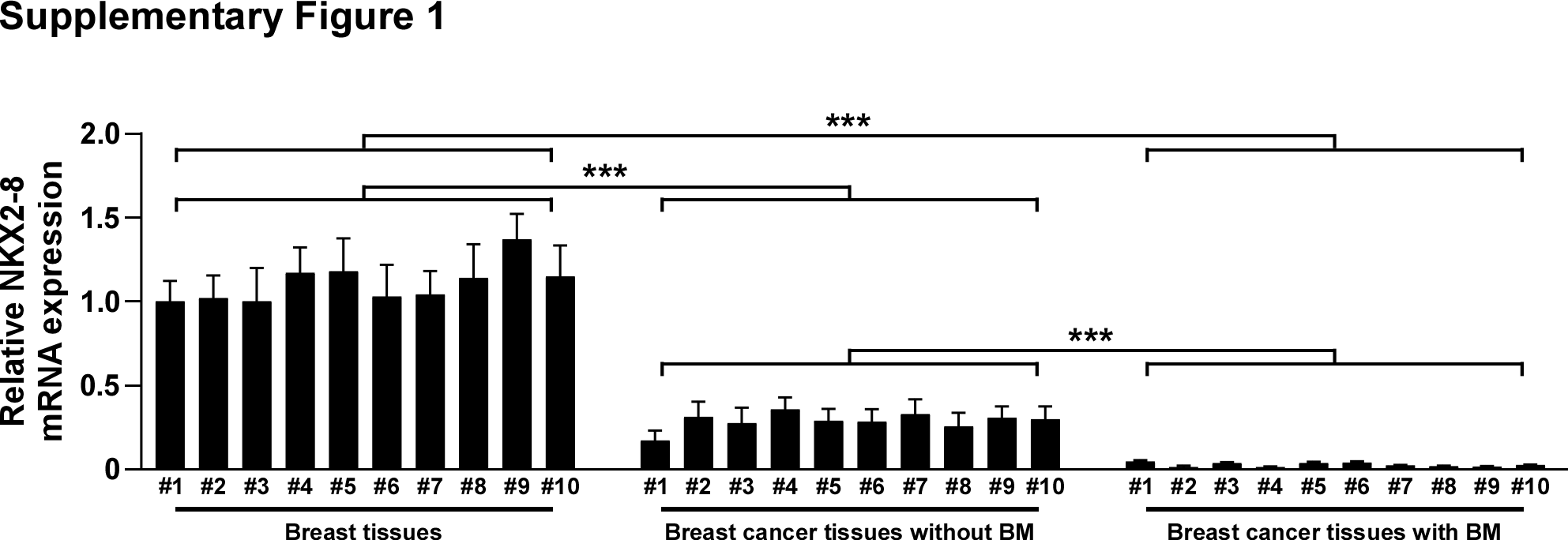


**Supplementary Figure 1. NKX2-8 is downregulated in breast cancer tissues.** Real-time PCR analysis of NKX2-8 expression in normal breast and breast cancer tissues with or without bone metastasis. GAPDH served as the loading control. *** *P* < 0.001.


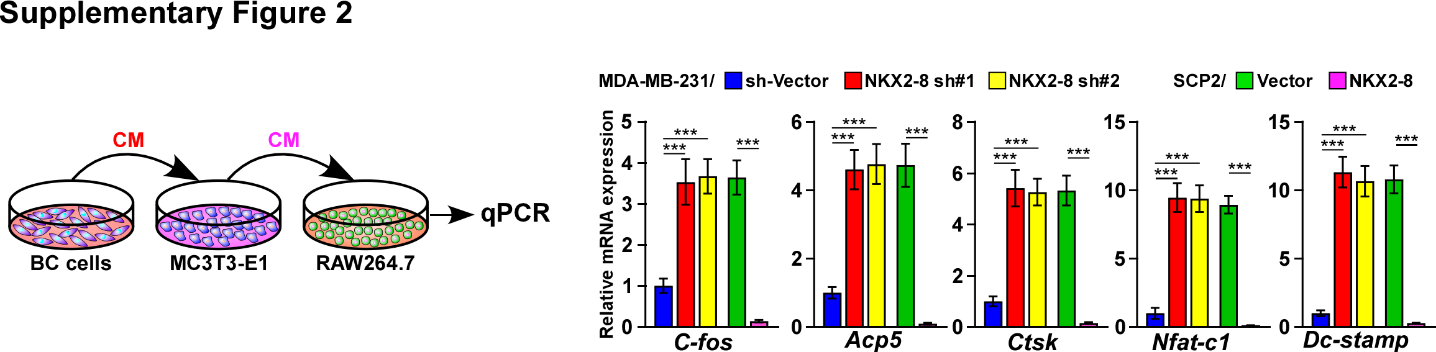


**Supplementary Figure 2. NKX2-8 regulates the expression of osteoclast differentiation marker genes.** Real-time PCR analysis of the mRNA level of osteoclast differentiation markers, including *C-fos*, *Acp5*, *Ctsk*, *Nfat-c1* and *Dc-stamp*, in RAW264.7 cells cultured with CM-SCP2/vector, or CM-SCP2/NKX2-8, or CM-MDA-MB-231/sh-Vector, or CM-MDA-MB-231/NKX2-8 sh#1, or CM-MDA-MB-231/NKX2-8 sh#2. *GAPDH* served as the loading control.


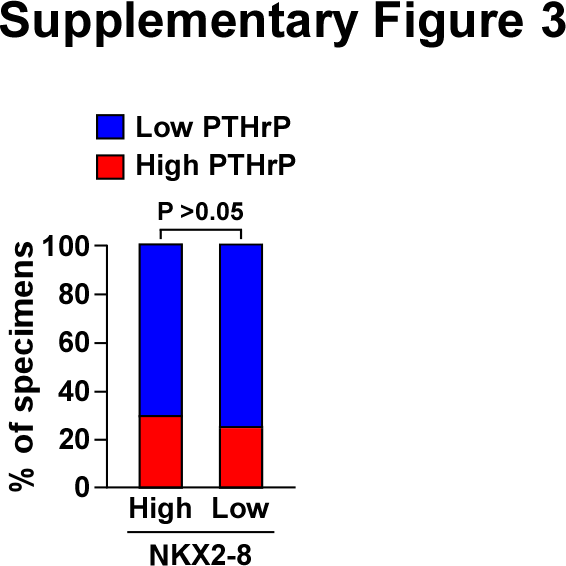


**Supplementary Figure 3. The expression of NKX2-8 is no correlative with expression of PTHrP in breast cancer tissues with metastasis to other organs.** Percentages of specimens showing low or high NKX2-8 expression relative to the levels of PTHrP in breast cancer tissues (n = 304) with other organs metastasis (n = 29).


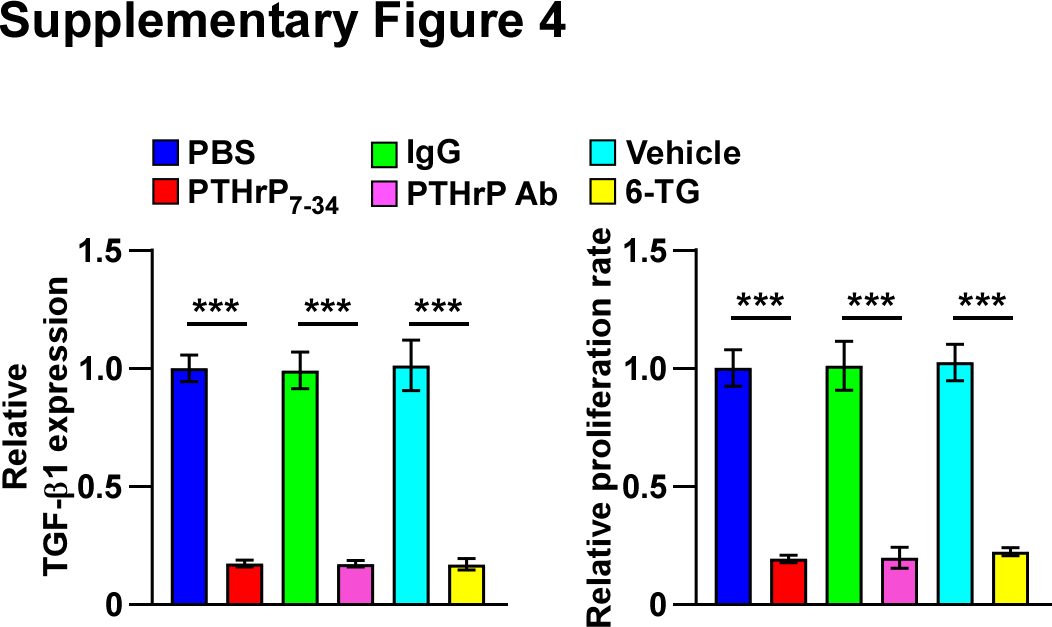


**Supplementary Figure 4. Targeting PTHrP inhibits NKX2-8 silencing-induced vicious cycle.** Left: ELISA analysis of TGF-β1 levels in CM from RAW 264.7 cells cultured onto the bone slice in the presence of indicated CM from breast cancer cells and treated with PBS or PTHrP7-34 or IgG or anti-PTHrP antibody or vehicle or 6-TG. Right: MTT assay analysis of proliferation rate of indicated cells from experiment in left panel. *** *P* < 0.001

## Supplementary Tables

**Supplementary table1.** Primers and Oligonucleotides used in this study.

| **Primer used for subcloning and plasmid construction** | | |
| --- | --- | --- |
| NKX2-8-1-86aa-up | gtgtcgtgaggattgggatccGCCATGGCCACCTCTGGA |  |
| NKX2-8-1-86aa-down | cttcatatgttcgaagaattcTCACAGATCCTCTTCAGAGATGAGTT |  |
| NKX2-8-87-140aa-up | gtgtcgtgaggattgggatccGCCATGCGGCGGGTGCTA |  |
| NKX2-8-87-140aa-down | cttcatatgttcgaagaattcTCACAGATCCTCTTCAGAGATGAGTT |  |
| NKX2-8-141-239aa-up | gtgtcgtgaggattgggatccGCCATGCGCGCTCGCGCT |  |
| NKX2-8-141-239aa-down | cttcatatgttcgaagaattcTCACAGATCCTCTTCAGAGATGAGTT |  |
| HDAC1-1-53aa-up | gtgtcgtgaggattgggatccGCCATGGCGCAGACGCAG |  |
| HDAC1-1-53aa-down | cttcatatgttcgaagaattcTCACTTATCGTCGTCATCCTTGTAA |  |
| HDAC1-54-325aa-up | gtgtcgtgaggattgggatccGCCATGTATCGCCCTCACAA |  |
| HDAC1-54-325aa-down | cttcatatgttcgaagaattcTCACTTATCGTCGTCATCCTTGTAA |  |
| HDAC1-326-482aa-up | gtgtcgtgaggattgggatccGCCATGAATGAGCTTCCATACA |  |
| HDAC1-326-482aa-down | cttcatatgttcgaagaattcTCACTTATCGTCGTCATCCTTGTAA |  |
| **Primer used for CAPTURE system** | | |
| PTHrP-sgRNA-1 | TATCTATACACTCCTCCCTG |  |
| PTHrP-sgRNA-2 | ACTGTAATAAACTGCCACTG |  |
| PTHrP-sgRNA-3 | CATGGGCTAGGCATTTGTGT |  |
| PTHrP-sgRNA-4 | GTTTAGTTGTTTGGTTCCAG |  |
| PTHrP-sgRNA-5 | GTTTGCTGTTTGGGTTAAGG |  |
| **Primers used for qPCR** | | |
| PTHrP-up | ATTTACGGCGACGATTCTTCC |  |
| PTHrP-down | GCTTGGAGTTAGGGGACACC |  |
| C-fos-up | CCGGGGATAGCCTCTCTTACT |  |
| C-fos-down | CCAGGTCCGTGCAGAAGTC |  |
| ACP5-up | GACTGTGCAGATCCTGGGTG |  |
| ACP5-down | GGTCAGAGAATACGTCCTCAAAG |  |
| NFATc1-up | CACCGCATCACAGGGAAGAC |  |
| NFATc1-down | GCACAGTCAATGACGGCTC |  |
| DCSTAMP-up | CGCTGCCTCCTGGATTATCAC |  |
| DCSTAMP-down | AAGCTCTTTGCCCTTAGGTTG |  |
| CTSK-up | ACACCCACTGGGAGCTATG |  |
| CTSK-down | GACAGGGGTACTTTGAGTCCA |  |
| **Primer used for ChIP-qPCR** | | |
| PTHrP-promoter-up | GCCCTGTTCCTGGAGAAGTC |  |
| PTHrP-promoter-down | ACTTGAGAGGAGGCTGTTGC |  |
| **Sequences mature sense of shRNA/siRNA** | | |
| NKX2-8 shRNA#1 | GCGCAGCCTTCTAGATTTACC |  |
| NKX2-8 shRNA#2 | GCTTGGGCCTTATTTGTATAT |  |
| PTHrP siRNA#1 | GCCAAGGCUAAUCCAAUUATT |  |
| PTHrP siRNA#2 | UUCACGGAGGCAUUGAAAUTT |  |
| HDAC1 shRNA#1 | TAGAGACCATAGTTGAGCAGC |  |
| HDAC1 shRNA#2 | GCCGGUCAUGUC⁃CAAAGUATT |  |

**Supplementary table2.** 34 proteins in Protein profiling of SCP2 vs. MDA-MB-231 cells.

| Uniprot ID\|Protein name | Description | 231 | 231 | 231 | SCP2 | SCP2 | SCP2 | Log2FoldChange | pvalue |
| --- | --- | --- | --- | --- | --- | --- | --- | --- | --- |
| Q9UHI8\|ATS1_HUMAN | A disintegrin and metalloproteinase with thrombospondin motifs 1 OS=Homo sapiens GN=ADAMTS1 PE=1 SV=4 | 1.79263 | 0.29882 | 1.62389 | 2.90109 | 4.06235 | 3.67687 | 1.517971426 | 0.01667 |
| P19883\|FST_HUMAN | Follistatin OS=Homo sapiens OX=9606 GN=FST PE=1 SV=2 | 2.42367 | 0.24498 | 0.89885 | 4.12929 | 3.15185 | 2.71723 | 1.486778721 | 0.04939 |
| P20809\|IL11_HUMAN | Nterleukin-11 OS=Homo sapiens GN=IL11 PE=1 SV=1 | 2.14297 | 1.30482 | 0.08883 | 2.85656 | 2.84308 | 3.65152 | 1.402771397 | 0.04131 |
| P29279\|CCN2_HUMAN | Connective tissue growth factor OS=Homo sapiens GN=CTGF PE=1 SV=2 | 1.78437 | 1.35944 | 0.61118 | 2.91412 | 3.11999 | 3.13889 | 1.288585132 | 0.00673 |
| P61073\|CXCR4_HUMAN | C-X-C chemokine receptor type 4 OS=Homo sapiens GN=CXCR4 PE=1 SV=1 | 1.62302 | 1.74035 | 0.35523 | 2.67446 | 3.4159 | 2.95672 | 1.282693176 | 0.02274 |
| P28562\|DUS1_HUMAN | Dual specificity protein phosphatase 1 OS=Homo sapiens GN=DUSP1 PE=1 SV=3 | 2.08798 | 1.47782 | 0.33075 | 2.84941 | 2.65618 | 3.46264 | 1.202628653 | 0.04122 |
| Q99457\|NP1L3_HUMAN | Nucleosome assembly protein 1-like 3 OS=Homo sapiens GN=NAP1L3 PE=2 SV=2 | 2.09225 | 0.66094 | 1.65324 | 3.20345 | 3.49054 | 3.31734 | 1.183948556 | 0.01235 |
| P19878\|NCF2_HUMAN | Neutrophil cytosol factor 2 OS=Homo sapiens GN=NCF2 PE=1 SV=2 | 1.84499 | 1.05521 | 0.78903 | 1.93896 | 3.48119 | 2.91392 | 1.175699345 | 0.04826 |
| P03956\|MMP1_HUMAN | Interstitial collagenase OS=Homo sapiens GN=MMP1 PE=1 SV=3 | 1.72522 | 1.36969 | 1.22438 | 3.59009 | 2.4385 | 3.65471 | 1.164704289 | 0.01331 |
| P12034\|FGF5_HUMAN | Fibroblast growth factor 5 OS=Homo sapiens GN=FGF5 PE=1 SV=4 | 2.57459 | 0.34555 | 1.66916 | 3.50545 | 3.19218 | 3.4346 | 1.142603576 | 0.04761 |
| Q93077\|H2A1C_HUMAN | Histone H2A type 1-C OS=Homo sapiens GN=HIST1H2AC PE=1 SV=3 | 2.00694 | 1.01905 | 1.43448 | 3.17717 | 3.50243 | 3.05298 | 1.125628133 | 0.00513 |
| O14508\|SOCS2_HUMAN | Suppressor of cytokine signaling 2 OS=Homo sapiens GN=SOCS2 PE=1 SV=1 | 2.34634 | 0.8068 | 1.72115 | 3.43972 | 3.50563 | 3.68018 | 1.124267634 | 0.01332 |
| P28562\|DUS1_HUMAN | Dual specificity protein phosphatase 1 OS=Homo sapiens GN=DUSP1 PE=1 SV=3 | 2.38451 | 1.14677 | 1.17698 | 3.82146 | 3.46924 | 2.95156 | 1.121267125 | 0.01836 |
| Q6FI13\|H2A2A_HUMAN | Histone H2A type 2-A OS=Homo sapiens GN=HIST2H2AA3 PE=1 SV=3 | 2.08722 | 1.0494 | 1.91617 | 3.8511 | 3.13581 | 3.75878 | 1.088606173 | 0.00839 |
| Q13308\|PTK7_HUMAN | Tyrosine-protein kinase-like 7 OS=Homo sapiens. GN=PTK7 PE=1 SV=2 | 2.04713 | 0.86557 | 1.37023 | 2.36731 | 2.98945 | 3.50418 | 1.04886037 | 0.03241 |
| Q9Y274\|SIA10_HUMAN | Type 2 lactosamine alpha-2,3-sialyltransferase OS=Homo sapiens GN=ST3GAL6 PE=1 SV=1 | 1.78097 | 1.2839 | 1.47317 | 3.25007 | 2.32725 | 3.68279 | 1.028959932 | 0.0208 |
| Q9Y676\|RT18B_HUMAN | 28S ribosomal protein S18b, mitochondrial OS=Homo sapiens GN=MRPS18B PE=1 SV=1 | 2.74124 | 1.09205 | 1.11102 | 3.61989 | 3.57409 | 2.81342 | 1.017226176 | 0.04955 |
| P53794\|SC5A3_HUMAN | Sodium/myo-inositol cotransporter OS=Homo sapiens GN=SLC5A3 PE=2 SV=2 | 1.39355 | 1.76892 | 1.68689 | 3.04981 | 3.48112 | 3.22654 | 1.008713927 | 0.00064 |
| P51636\|CAV2_HUMAN | Caveolin-2 OS=Homo sapiens GN=CAV2 PE=1 SV=2 | 3.61794 | 3.52357 | 2.4819 | 1.38829 | 2.08903 | 1.27154 | -1.018967241 | 0.02168 |
| P08697\|A2AP_HUMAN | Alpha-2-antiplasmin OS=Homo sapiens GN=SERPINF2 PE=1 SV=3 | 3.80012 | 3.14129 | 2.94445 | 1.54286 | 1.52092 | 1.67887 | -1.059670799 | 0.00288 |
| A1X283\|SPD2B_HUMAN | SH3 and PX domain-containing protein 2B OS=Homo sapiens GN=SH3PXD2B PE=1 SV=3 | 3.85821 | 2.73582 | 2.87943 | 1.53659 | 1.59031 | 1.40468 | -1.063877483 | 0.00992 |
| Q96HU1\|SGSM3_HUMAN | Small G protein signaling modulator 3 OS=Homo sapiens GN=SGSM3 PE=1 SV=1 | 2.48574 | 3.40222 | 2.63706 | 1.76871 | 1.63297 | 0.63712 | -1.077778567 | 0.03047 |
| Q14155\|ARHG7_HUMAN | Rho guanine nucleotide exchange factor 7 OS=Homo sapiens GN=ARHGEF7 PE=1 SV=2 | 2.79237 | 2.36383 | 3.0853 | 0.8599 | 2.19711 | 0.8444 | -1.07891069 | 0.0431 |
| Q12774\|ARHG5_HUMAN | Rho guanine nucleotide exchange factor 5 OS=Homo sapiens GN=ARHGEF5 PE=1 SV=3 | 2.84086 | 2.83871 | 4.25944 | 1.13148 | 2.19297 | 1.00373 | -1.199337447 | 0.03652 |
| P20908\|CO5A1_HUMAN | Collagen alpha-1(V) chain OS=Homo sapiens GN=COL5A1 PE=1 SV=3 | 3.54648 | 2.40264 | 4.2606 | 0.70235 | 2.17503 | 1.54662 | -1.206519542 | 0.04887 |
| P29034\|S10A2_HUMAN | Protein S100-A2 OS=Homo sapiens GN=S100A2 PE=1 SV=3 | 3.12887 | 2.66234 | 3.83957 | 0.89573 | 2.04438 | 1.17961 | -1.225106365 | 0.01947 |
| Q96QB1\|RHG07_HUMAN | Rho GTPase-activating protein 7 OS=Homo sapiens GN=DLC1 PE=1 SV=4 | 2.76162 | 3.46528 | 3.36055 | 1.07133 | 1.64115 | 1.32828 | -1.246519951 | 0.00252 |
| Q9NS25\|SPNXB_HUMAN | Sperm protein associated with the nucleus on the X chromosome B1 OS=Homo sapiens OX=9606 GN=SPANXB1 PE=1 SV=2 | 3.21861 | 4.1385 | 3.79598 | 1.07993 | 1.97902 | 1.5209 | -1.284070948 | 0.00421 |
| P78423\|X3CL1_HUMAN | Fractalkine OS=Homo sapiens GN=CX3CL1 PE=1 SV=1 | 2.94116 | 3.62309 | 3.39709 | 1.05932 | 1.58422 | 1.41566 | -1.295141371 | 0.00148 |
| P01009\|A1AT_HUMAN | Alpha-1-antitrypsin OS=Homo sapiens GN=SERPINA1 PE=1 SV=3 | 3.57637 | 4.14037 | 3.79468 | 1.04661 | 1.3146 | 2.24669 | -1.320885245 | 0.00449 |
| P33764\|S10A3_HUMAN | Protein S100-A3 OS=Homo sapiens GN=S100A3 PE=1 SV=1 | 2.84763 | 3.14188 | 3.52833 | 1.23163 | 1.70708 | 0.7145 | -1.381467953 | 0.00493 |
| P23297\|S10A1_HUMAN | Protein S100-A1 OS=Homo sapiens GN=S100A1 PE=1 SV=2 | 2.7502 | 2.7044 | 3.06089 | 1.16628 | 1.45514 | 0.45668 | -1.468044879 | 0.00463 |
| O15438\|MRP3_HUMAN | Canalicular multispecific organic anion transporter 2 OS=Homo sapiens GN=ABCC3 PE=1 SV=3 | 3.9044 | 4.73868 | 2.86078 | 1.34744 | 1.03392 | 1.46006 | -1.582404088 | 0.0102 |
| O15522\|NKX28_HUMAN | Homeobox protein Nkx-2.8 OS=Homo sapiens GN=NKX2-8 PE=2 SV=2 | 3.86136 | 3.21293 | 3.12556 | 0.98905 | 1.04666 | 0.93135 | -1.781445568 | 0.0005 |

**Supplementary table3.** Potential trans-regulatory factors were enriched on the PTHrP promoter in MDA-MB-231 cells.

| **Accession** | **Description** | **-10lgP** | **#Peptides** | **#Unique** |
| --- | --- | --- | --- | --- |
| O15522\|NKX28_HUMAN | Homeobox protein Nkx-2.8 OS=Homo sapiens GN=NKX2-8 PE=2 SV=2 | 338.42 | 51 | 37 |
| Q13547\|HDAC1_HUMAN | Histone deacetylase 1 OS=Homo sapiens GN=HDAC1 PE=1 SV=1 | 296.57 | 32 | 29 |
